# VGSC-DB: an online database of voltage-gated sodium channels

**DOI:** 10.1186/s13321-022-00655-y

**Published:** 2022-11-01

**Authors:** Gaoang Wang, Jiahui Yu, Hongyan Du, Chao Shen, Xujun Zhang, Yifei Liu, Yangyang Zhang, Dongsheng Cao, Peichen Pan, Tingjun Hou

**Affiliations:** 1 Innovation Institute for Artificial Intelligence in Medicine of Zhejiang University, Zhejiang University, Hangzhou, 310058 Zhejiang China; 2 Xiangya School of Pharmaceutical Sciences, Central South University, Changsha, 410004 Hunan China

**Keywords:** Voltage-gated sodium channels, VGSC-DB, Drug discovery, Open-source database, Computer-aided drug design

## Abstract

As an important member of ion channels family, the voltage-gated sodium channel (VGSC/Na_v_) is associated with a variety of diseases, including epilepsy, migraine, ataxia, etc., and has always been a hot target for drug design and discovery. Many subtype-selective modulators targeting VGSCs have been reported, and some of them have been approved for clinical applications. However, the drug design resources related to VGSCs are insufficient, especially the lack of accurate and extensive compound data toward VGSCs. To fulfill this demand, we develop the Voltage-gated Sodium Channels Database (VGSC-DB). VGSC-DB is the first open-source database for VGSCs, which provides open access to 6055 data records, including 3396 compounds from 173 references toward nine subtypes of Na_v_s (Na_v_1.1 ~ Na_v_1.9). A total of 28 items of information is included in each data record, including the chemical structure, biological activity (IC_50_/EC_50_), target, binding site, organism, chemical and physical properties, etc. VGSC-DB collects the data from small-molecule compounds, toxins and various derivatives. Users can search the information of compounds by text or structure, and the advanced search function is also supported to realize batch query. VGSC-DB is freely accessible at http://cadd.zju.edu.cn/vgsc/, and all the data can be downloaded in XLSX/SDF file formats.

**Graphical Abstract:**

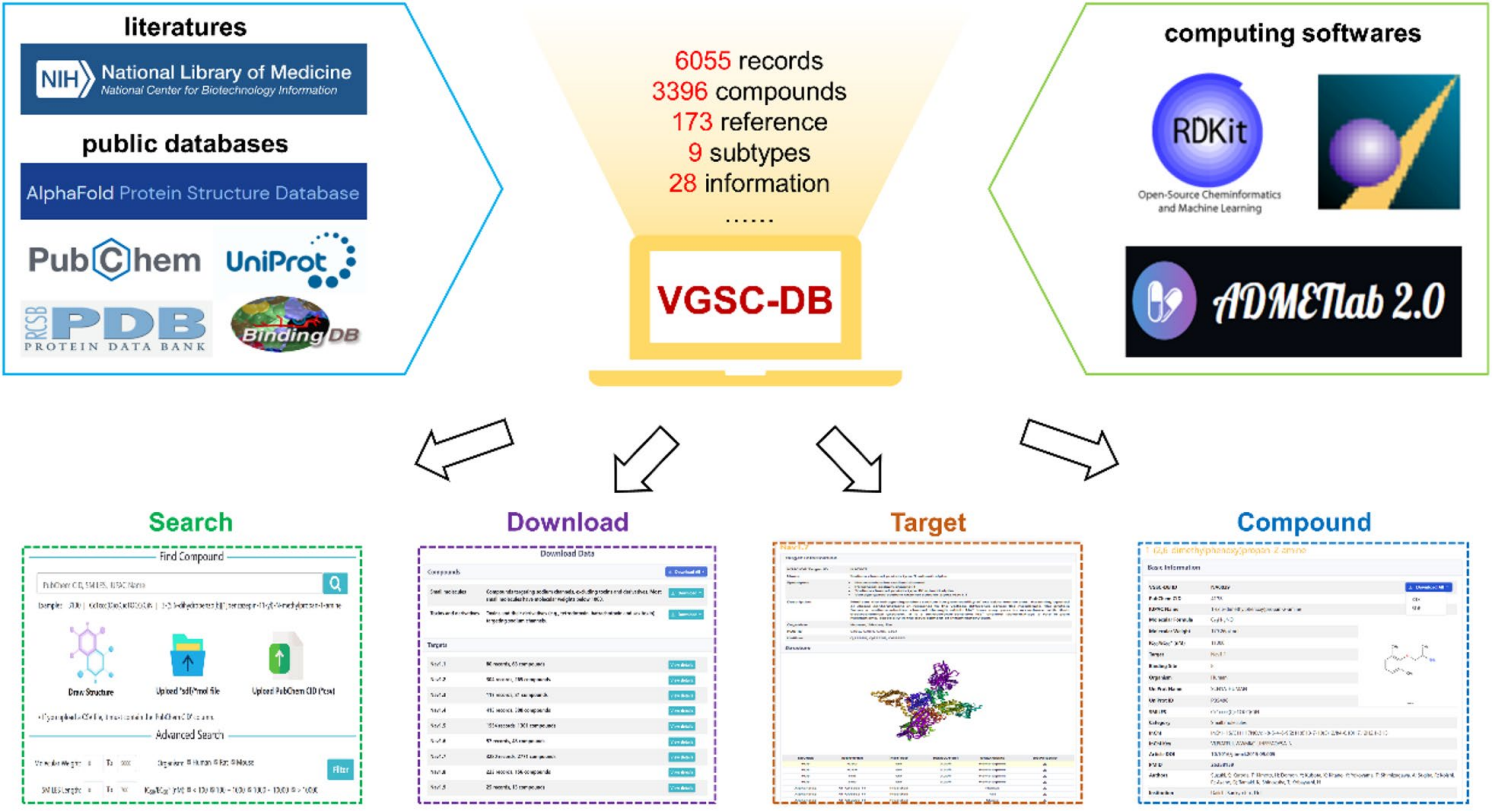

## Introduction

Voltage-gated sodium channels (VGSCs/Na_v_s) are the key elements for the generation and conduction of action potentials (APs), which control the entry and exit of sodium ions in cells. To date, nine Na_v_1 subtypes (Na_v_1.1 ~ Na_v_1.9) have been found in mammals, and each subtype has its own unique functions [1]. Na_v_1.1, Na_v_1.2, Na_v_1.3 and Na_v_1.6 are primarily expressed in the central nervous system (CNS), Na_v_1.4 is predominantly expressed in skeletal muscle, Na_v_1.5 plays a critical role in heart muscle regulation, and Na_v_1.7 to Na_v_1.9 generally regulate the function of peripheral nervous system (PNS) [2–5]. Due to the significance of its physiological functions, VGSC is the focus of biological structure research. In mammals, each VGSC is formed by one α subunit (~ 2000 amino acids) and one or two β subunits. Generally, the α subunit constitutes the main part of VGSC, and the β subunit mainly plays a regulatory role [6]. The α subunit contains four domains (DI ~ DIV), and each domain is formed by six α-helices transmembrane segments (S1 ~ S6) linked by extracellular or intracellular loops. The S1 ~ S4 segments form the voltage-sensing domain (VSD), which can sense the change of external voltage and adjust the opening of channel when the membrane is depolarized [7]. Segments S5, S6 and the extracellular connecting pore-loops (P-loops) form the central pore and the selectivity filter (SF), which are responsible for ion selectivity and permeation. Structural analysis results confirm that VGSC has at least three physiological states, namely activated (open), inactivated (closed) and resting (closed) [8]. VGSCs cycle through three states in a specific sequence, i.e., open-closed-deactivated cycle [9]. Up to now, there are six experimentally determined structures of human-derived VGSCs resolved (i.e., hNa_v_1.1 [10], hNa_v_1.2 [11], hNa_v_1.3 [12], hNa_v_1.4 [13], hNa_v_1.5 [14, 15] and hNa_v_1.7 [16]) which greatly promoted the progress of drug design and discovery targeting VGSCs.

At present, many drugs targeting VGSCs have been approved and used in clinic, such as lamotrigine, carbamazepine and phenytoin [17]. However, serious toxic and side effects remains to be a major problem since these drugs are non-subtype-selective modulators of VGSCs [18, 19]. Therefore, development of subtype-selective molecules targeting VGSCs has become a hot spot in drug design. In recent years, several lead compounds with potent activity and high subtype selectivity have been studied in clinical trials (e.g., PF-05089771 [20], ICA-121431 [21], NBI-921352 [22]). These subtype-selective molecules can selectively inhibit a certain Na_v_ subtype by binding to the VSDIV domain [23]. Despite great efforts have been made, there are still no subtype-selective drugs available on the market. Thus, development of next generation subtype-selective small-molecule modulators targeting VGSCs is still quite urgent.

However, the drug design resources of VGSCs are limited. For drug discovery, researchers need precise and extensive data resources, particularly compound information. Numerous modelling and drug design methods (e.g., virtual screening, molecular generation, structural modification) necessitate a plethora of compound information. Although some comprehensive databases, such as PubChem [24], ChEMBL [25] and BindingDB [26], collect several relevant data on VGSCs, the compound information for VGSCs in these databases remains limited and redundant. For instance, these databases do not include information on the binding sites of compounds, and many of the data lack the information about biological activities. Consequently, there is a lack of a professional and comprehensive database for VGSCs.

**Fig. 1:**
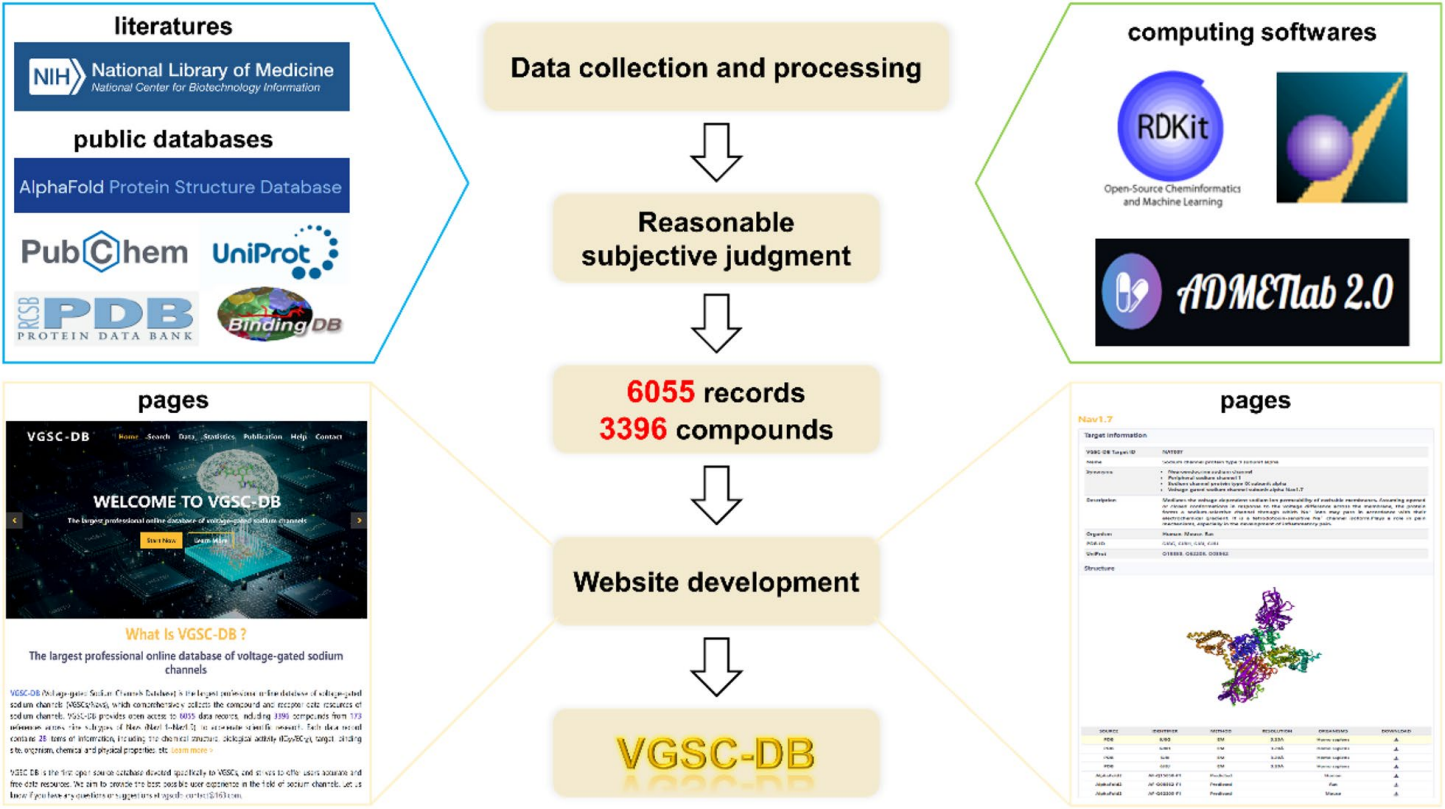
The basic framework of VGSC-DB

In this work, we develop the Voltage-gated Sodium Channels Database (VGSC-DB) (Fig. 1). As far as we are aware, VGSC-DB is the first open-source and largest professional online database of VGSCs, which collects the compound and receptor data resources of sodium channels exhaustively. VGSC-DB provides 6055 data records, corresponding to 3396 compounds and 173 references, covering 9 subtypes of Na_v_s (Na_v_1.1 ~ Na_v_1.9). Each data record contains 28 items of information, including the chemical structure, biological activity (IC_50_/EC_50_), target, binding site, organism, chemical and physical properties, and so on. Each data has been manually validated and is supported by the corresponding reference. VGSC-DB can be queried with two general approaches: the text-based (i.e., input PubChem CID and IUPAC Name) and structure-based search (i.e., input SMILES, draw structure, upload SDF/MOL files). In addition, VGSC-DB supports advanced searches, where a large number of compounds can be searched by selecting molecular weight, SMILES length, organism, and IC_50_/EC_50_. VGSC-DB collects data for small molecules, toxins and various derivatives including natural toxins (e.g., tetrodotoxin, batrachotoxin, saxitoxin) and their derivatives, and the majority are unique. All the data in VGSC-DB are available for downloading as XLSX/SDF file formats, and can be used for model construction and structure–activity relationship analysis. In summary, VGSC-DB provides accurate and extensive data of VGSCs, which would become a valuable resource for VGSC drug design and discovery.

## Materials and methods

### Data collection and processing

The data of VGSC-DB were collected from literatures and public databases. The literatures were searched in the PubMed database using the keywords of ‘(sodium channel) AND ((modulator) OR (molecular))’, and the retrieval scope was the article title and abstract. We manually collected and checked the important compound information from the literatures, including the structure, biological activity (IC_50_/EC_50_), target, binding site, etc. We also collated the compound and receptor information in multiple well-known databases, including PubChem, PubMed, BindingDB, UniProt [27], PDB [28] and AlphaFold Protein Structure Database [29]. It was worth noting that all data in VGSC-DB were collected based on peer-reviewed literatures rather than patents to ensure the high reliability of the information.

For compound information, each data record with unique VGSC-DB ID contained 28 items of information. Some toxins and derivatives were not included in the PubChem database, so the PubChem CID numbers of these compounds were labeled by ‘none’. The 2D structures of compounds were generated by Indigo software [30] as PNG format. The biological activity was represented by half maximal inhibitory concentration (IC_50_) and half maximal effective concentration (EC_50_), and the default unit was nanomolar (nM). To help users distinguish the biological activity, the EC50 value is indicated by ‘*’ in the upper right corner of the number. The regional division standard of the binding site was determined based on the summary of many literature reports [2, 31, 32]. The information of IC_50_/EC_50_ value, target, binding site and organism were derived from the references. The information of IUPAC name, molecular formula and molecular weight came from the PubChem and BindingDB databases. The SMILES of all compounds were calculated by RDKit software (http://www.rdkit.org/) to unify the standard, and the InChI and InChI Key were calculated based on SMILES by using RDKit. The information of article DOI, PMID, authors and author affiliation were collected from the PubMed database. In addition, eight important physicochemical properties related to drug-likeness were predicted and integrated into VGSC-DB. The numbers of heavy atom, ring, hydrogen bond acceptor, hydrogen bond donor and rotatable bond were calculated by RDKit, and the log*P*, log*S* and log*D* were calculated by ADMETlab 2.0 software [33]. The 2D SDF files were generated by RDKit, and molecules were washed in MOE software (https://www.chemcomp.com/). The detailed information of compounds was provided in XLSX files.

The VGSC-DB Target ID was also a unique code in the database, which was related to the Na_v_ subtype but not organism. The name, synonyms, description and UniProt ID of the target came from the UniProt database with some statements modified. The experimental and predicted target structures derived from the PDB and AlphaFold Protein Structure Database, and these structures could be downloaded in PDB format. All files in VGSC-DB could be downloaded for free.

We next processed and optimized the data based on the above information. There were huge structural differences between small molecules and toxins. Therefore, we firstly divided all compounds into small molecules and toxin derivatives according to their structures and molecular weight. The group of small molecules did not contain toxins, and users could make better use of these data for specific studies. Secondly, we used multiple data entries to record the information of compounds separately. For VGSCs, a compound could target different subtypes and the data might be generated under different experimental conditions or tested by various methods. VGSC-DB summarized the data of a compound in separate records based on the information of targets, experimental methods, organisms, etc. Users were able to view the details of each record to find pertinent data. Besides, the activity data were the basis for quantitative and qualitative analysis. In VGSC-DB, each record must include the information of biological activity, and the compounds without explicit data records were not collected into the database. Notably, studies from different groups might have opposite conclusions, and the controversial data would bring a lot trouble to users. And some common-sense contents (e.g., the binding site of tetrodotoxin) were not mentioned in many literatures. We therefore manually validated and modified those data records based on the timeliness and authority of the research. In brief, we optimized and adjusted the data based on objective facts to help users get the best experience.

### Online database implementation

The website was built with the Django 3.2.2 framework by using Python 3.6.12. All the data from the database was stored in SQLite. The text-based search strings were PubChem CID and IUPAC name. Users could achieve quick search for a batch of molecules by uploading a CSV file containing multiple PubChem CIDs. Users could input SMILES strings, draw a molecule within the ChemDoodle editor [34] or upload a SDF/MOL file to realize the structure-based search. The 3D structures of protein targets were displayed by using 3Dmol.js [35], which was an object-oriented and WebGL-based JavaScript library for online protein visualization. The information of the compound was displayed in table through DataTables.js. The highcharts.js was embedded into the website to display the statistics of the database. All technical operations of VGSC-DB were in line with the specifications.

## Results and discussions

### Statistics in VGSC-DB

**Fig. 2:**
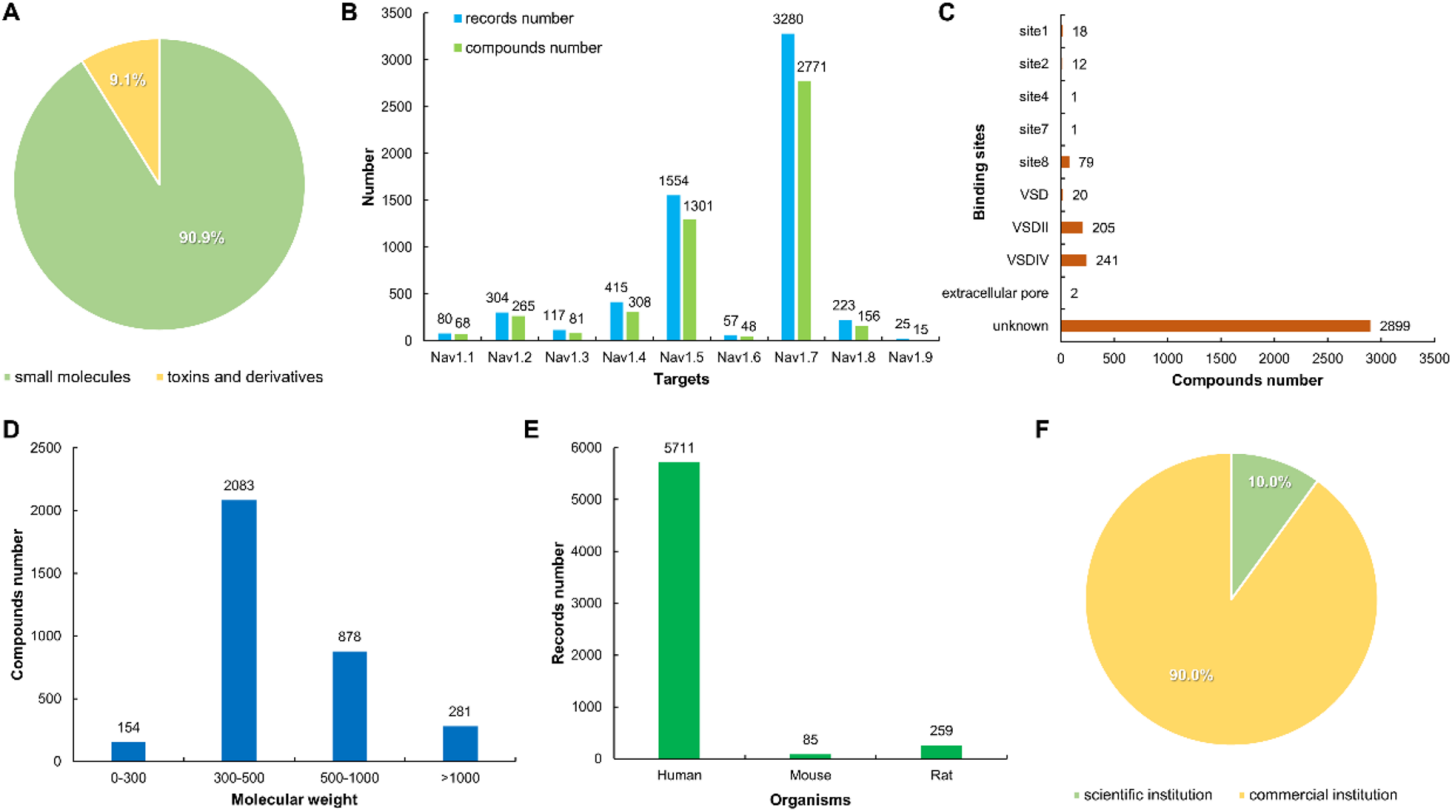
Statistics in VGSC-DB. **A** The proportion of molecular types in the database. **B** The distribution of data records and compounds in the database. **C** The distribution of binding sites of compounds. **D** The distribution of molecular weight of compounds. **E** The distribution of organisms of data records. **F** The proportion of data records sources

The compounds in VGSC-DB are divided into two categories: small molecules (90.9%), and toxin derivatives (9.1%) (Fig. 2A). VGSC-DB uses multiple data entries to record compound information to provide the best user experience. There are 6055 data records in VGSC-DB with 3396 compounds collected from 173 references, covering 9 targets from Na_v_1.1 to Na_v_1.9. Each data record contains 28 items of information, which comprehensively summarizes and shows the properties of VGSC modulators.

Figure 2B shows the number of compounds or records collected for each subtype. Na_v_1.7 compounds account for a significant proportion of VGSC-DB, which includes 2771 compounds and 3280 records. The numbers of compounds and records for Na_v_1.5 subtype are 1301 and 1554, respectively. The total records of both Na_v_1.7 and Na_v_1.5 comprise more than 80% of all the database, indicating the importance of these two targets for drug design. The number of the records for the other subtypes is relatively small, specifically as follows: Na_v_1.4 (308 compounds and 415 records), Na_v_1.2 (265 compounds and 304 records), Na_v_1.8 (156 compounds and 223 records), Na_v_1.3 (81 compounds and 117 records), Na_v_1.1 (68 compounds and 80 records), Na_v_1.6 (48 compounds and 57 records) and Na_v_1.9 (15 compounds and 25 records). The unbalanced subtype distribution of the reported compounds indicates that the research on VGSC needs further exploration, and the design of inhibitors targeting those ‘cold’ subtype is still in urgent need for innovative drug development. Bioactivity of the compounds is one of the most important data in the database (Table 1). Highly active molecules with  IC_50_/EC_50 _< 100 nM account for a relatively low proportion of the database (nearly 22.9%). The percentages of compounds with moderate bioactivity (IC_50_/EC_50_ = 100 ~ 1000 nM and 1000 ~ 10,000 nM) are 25.4% and 28.1%, respectively. Compounds with low activity (IC_50_/EC_50_ > 10,000 nM) comprise about 23.6% of all the molecules in VGSC-DB. Nearly 83.1%% of the highly active compounds are associated with Na_v_1.7, which reconfirms the important role of Na_v_1.7 in VGSC drug design. Of note, the bioactivity of a compound may vary greatly under different experimental conditions (e.g., cell, organism, stimulation voltage), so the detailed information is also made available to the users for personalized studies.

**Table 1:** The distribution of biological activity (IC_50_/EC_50_) of data records

Targets	Number of records in IC_50_/EC_50 _ (nM) range				
	0–10	10–100	100–1000	1000–10,000	> 10,000
Na_v_1.1	4	8	13	30	25
Na_v_1.2	22	31	61	120	70
Na_v_1.3	2	7	9	16	83
Na_v_1.4	4	27	79	209	96
Na_v_1.5	2	36	154	556	806
Na_v_1.6	6	6	18	12	15
Na_v_1.7	371	782	1122	725	280
Na_v_1.8	24	55	81	29	34
Na_v_1.9	–	–	1	3	21
Total	435	952	1538	1700	1430

Due to the structural complexity of VGSCs, it is difficult to determine the binding site of a VGSC modulator (Fig. 2C). The binding site information of only 579 compounds (17.0% of all the molecules in VGSC-DB) was collected since this information is not mentioned in the literature for most molecules. Among the 579 compounds with indicated binding sites, most (446 molecules) bind to the second VSD (VSDII) and VSDIV domain, and users can download these data for model analysis. Recently, the fourth VSD (VSDIV) was identified as a new binding domain, and several compounds targeting this domain are being studied in clinical trials [23].

In VGSC-DB, the molecular weights (MW) of most molecules (2237 molecules) are under 500, and 2083 compounds have MW in the range of 300 to 500. The proportion of compounds having MW above 500 is about 34.1%, with 25.9% in the range of MW from 500 to 1000. (Fig. 2D). In all data records, there are 5711 human data (94.3%), 259 rat data (4.3%) and 85 mouse data (1.4%) (Fig. 2E). Notably, only 10.0% data records came from the scientific institution (e.g., university and research laboratory), and the vast majority of the data were collected from the commercial institutions (e.g., pharmaceutical company and commercial laboratory) (Fig. 2F).

### Search and download of database

**Fig. 3:**
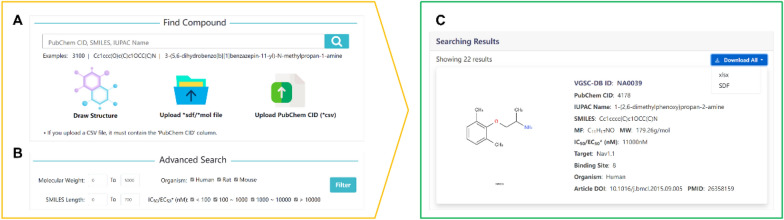
The search page and result page of VGSC-DB. **A** The text-based and structure-based search engine. **B** The advanced search engine. **C** Example of the searching results page

VGSC-DB provides users with accurate and efficient search function. There are two data query approaches: the text-based and structure-based search (Fig. 3A). Users can input the PubChem CID and IUPAC name for compound retrieval. For batch retrieval requirements, users can upload a CSV file containing the PubChem CIDs, and the database will retrieve all results. The name of the column containing the PubChem CID in the CSV file must be ‘PubChem CID’. Besides, users can input SMILES, upload a SDF/MOL file or draw the molecule structure within the ChemDoodle editor to realize structure-based search. After search is completed, it will jump to the ‘Searching Result Page’. The search results are displayed in the form of small cards, including the VGSC-DB ID, structure, SMILES, IC_50_/EC_50_, target, article DOI and the other brief information of compounds (Fig. 3C). Users can quickly view the basic information, and click the ‘VGSC-DB ID’ to jump to the ‘Compound Page’ to view more detailed information of compounds. The number of the query results is displayed on top of the ‘Searching Result Page’, and a Scroll-To-Top button at the bottom of the page allows quick return to the top. Moreover, VGSC-DB provides the advanced search function (Fig. 3B). Users can set the molecular weight, organism, SMILES length and IC_50_/EC_50_ values to quickly screen a large number of compounds. And the searched molecules can be downloaded in batch in XLSX/SDF file formats.

**Fig. 4:**
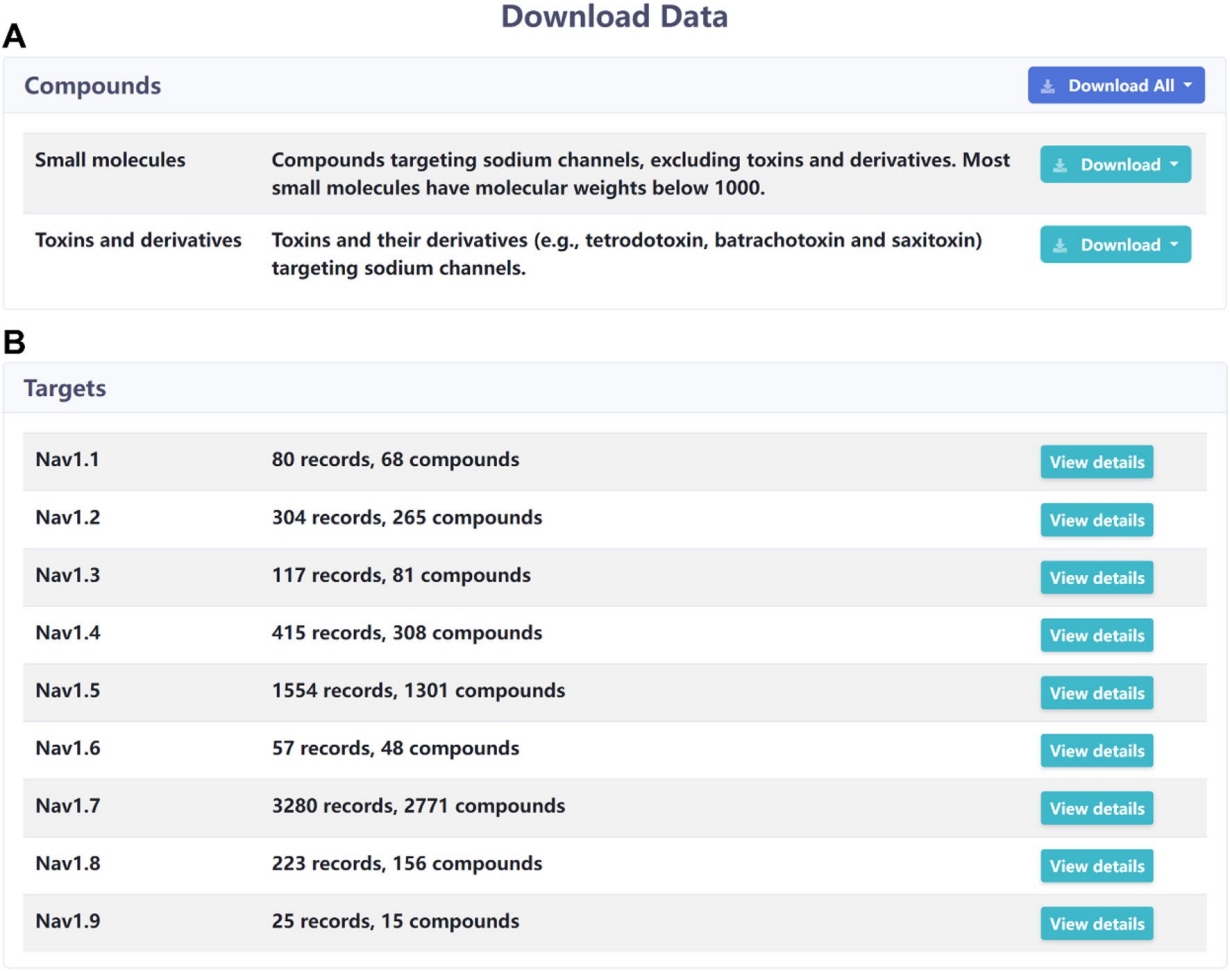
The data download page of VGSC-DB. **A** The compounds data available for download. **B** The target data of nine Nav subtypes

VGSC-DB provides a fast data download page (Fig. 4). This page is divided into two parts: compounds and targets. For compounds, users can download all compounds at once and download small molecules or toxins and derivatives by classification. All molecules are provided in XLSX/SDF file formats. In the lower part of the page, there are nine targets listed, i.e., Na_v_1.1 ~ Na_v_1.9. Users can click the ‘View details’ bottom to jump to the ‘Target Page’ to view more information.

### Target and compound information

**Fig. 5:**
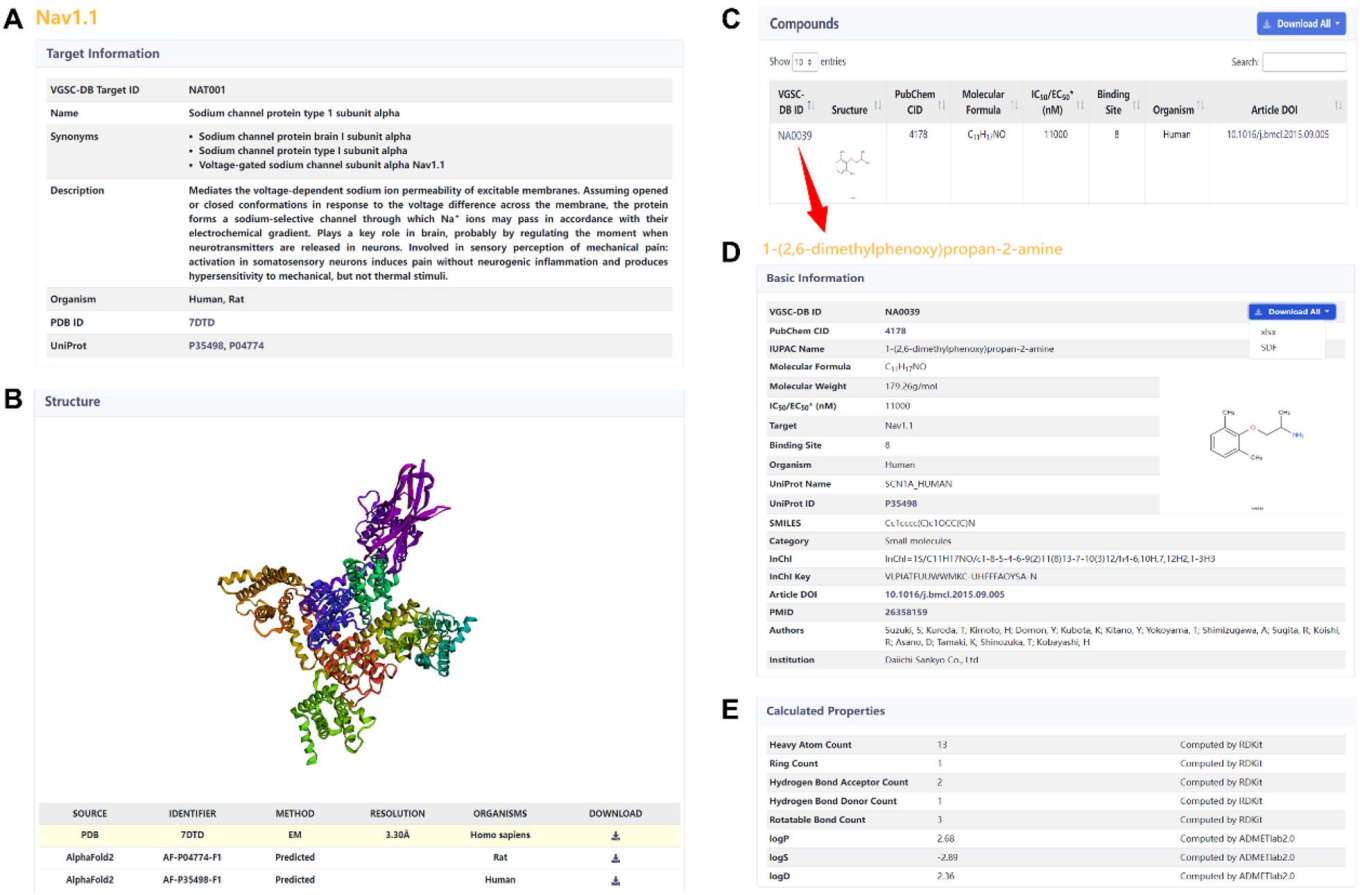
The target page and compound page of VGSC-DB. **A** The target information of Nav1.1. **B** The 3D protein structure display window. **C** The table of compounds targeting this protein subtype. **D** The detailed information of NA0039. **E** The predicted physical and chemical properties of NA0039

The ‘Target Page’ and ‘Compound Page’ contain the main information of VGSC-DB. In the ‘Target Page’, there are three parts: target information, structure and compounds (Fig. 5A, B, C). The target information contains the target ID, name and synonym, description, organism and PDB/UniProt ID of protein. Users can click the PDB/UniProt ID to jump to the corresponding pages. The 3D stereoscopic structures of protein are displayed in the window, and users can click the protein to rotate and zoom in/out. These structures come from the PDB and AlphaFold Protein Structure Database, and all proteins can be downloaded freely in PDB file format. The page shows the source, identifier, method, resolution and organism information to help users quickly distinguish these structures. All molecules targeting the specific protein subtype are listed in a table, which contains VGSC-DB ID, structure, PubChem CID, molecular formula, IC_50_/EC_50_, binding site, organism and article DOI. Users can click the VGSC-DB ID to jump to the ‘Compound Page’, and click the article DOI to view the literature. The table supports text retrieval and sorting functions, and users can switch pages and view the information of all the compounds. All compound data can be downloaded for free in XLSX/SDF file formats.

The ‘Compound Page’ is divided into two parts: basic information and calculated properties (Fig. 5D, E). In this page, the detailed information of compounds is shown, such as IUPAC name, target, SMILES, InChI, authors, and so on. Users can find almost all useful information of the compound, and click the four links (i.e., PubChem CID, UniProt ID, article DOI and PMID) to view more details. Moreover, we used the RDKit and ADMETlab2.0 software to calculate the physical and chemical properties of molecules, which provide clues for further analysis and drug design. All data of a compound also can be freely downloaded in XLSX/SDF file formats.

## Conclusion

Data is the foundation of drug design and discovery. Since the first human VGSCs structure (hNa_v_1.4 [13]) was reported, the drug design of VGSCs has advanced and a significant amount of compound data has demonstrated accelerated growth. Computational approaches are being used more and more in VGSC drug discovery as a result of advances in technology and algorithms. Compared with traditional experimental methods that explore the structure–activity relationship of compounds, computer-aided drug design (CADD) has developed a variety of methods (e.g., virtual screening, quantitative structure–activity relationship, artificial intelligence modeling) for accurate and rapid molecular design and generation. These approaches are increasingly driving drug discovery and enhancing data use. For instance, Sadybekov et al*.* developed a modular synthon-based approach, i.e., V-SYNTHES, to perform the structure-based screening in a large database that contained more than 11 billion compounds [36]. The V-SYNTHES method demonstrates a superior lead compound discovery hit rate and has a super-large data processing capacity unmatched by conventional techniques. The new models represented by V-SYNTHES not only improve the data processing ability, but also are the inevitable results of the emergence of a large number of high-quality data resources. The need for efficient and high-quality data resources is therefore greater than ever.

VGSC-DB, the first open-source database dedicated to VGSCs, aims to provide free data resource for VGSC research and drug discovery. We make great efforts to find and gather information for this database from all relevant public databases and publications. To make the data easier for users to access and use, we optimize the database in various ways. We hope that the VGSC-DB can provide convenience for users, and we also expect users can give us more feedback to help us improve. However, the database still has some limitations. Firstly, many compounds are not collected because the exact biological activity values are not available. Secondly, the website's pages and features still require further optimization. Besides, the data included in the database is still incomplete due to numerous constraints imposed by different data sources. Furthermore, some recent reported compounds are changing the previous classification rules. For instance, Wang et al*.* reported a compound that bound to the intracellular C-terminal domain of Na_v_1.6, suggesting that some modulators might bind to the allosteric sites of Na_v_ channel [37]. All in all, we will keep mining the original data and expanding the database, and strive to develop some computational capabilities including molecular generation and virtual screening models. We believe that VGSC-DB will serve as an important resource and a powerful tool for studying VGSC and further drug design of innovative VGSC modulators.

## References

[1] de Lera Ruiz M, Kraus RL (2015). Voltage-gated sodium channels: structure, function, pharmacology, and clinical indications. J Med Chem.

[2] Xu L, Ding X, Wang T, Mou S, Sun H, Hou T (2019). Voltage-gated sodium channels: structures, functions, and molecular modeling. Drug Discov Today.

[3] Hodgkin AL, Huxley AF (1945). Resting and action potentials in single nerve fibres. J Physiol.

[4] Hodgkin AL, Huxley AF (1952). A quantitative description of membrane current and its application to conduction and excitation in nerve. J Physiol.

[5] Ahern CA, Payandeh J, Bosmans F, Chanda B (2016). The hitchhiker's guide to the voltage-gated sodium channel galaxy. J Gen Physiol.

[6] Brackenbury WJ, Isom LL (2011). Na channel beta subunits: overachievers of the Ion channel family. Front Pharmacol.

[7] Xiao J, Bondarenko V, Wang Y (2021). Regulation and drug modulation of a voltage-gated sodium channel: pivotal role of the S4–S5 linker in activation and slow inactivation. Proc Natl Acad Sci USA.

[8] Catterall WA, Goldin AL, Waxman SG (2005). International Union of Pharmacology. XLVII. Nomenclature and structure-function relationships of voltage-gated sodium channels. Pharmacol Rev.

[9] Diaz-Garcia A, Varela D (2020). Voltage-Gated K(+)/Na(+) channels and scorpion venom toxins in cancer. Front Pharmacol.

[10] Pan X, Li Z, Jin X (2021). Comparative structural analysis of human Nav1.1 and Nav1.5 reveals mutational hotspots for sodium channelopathies. Proc Natl Acad Sci USA.

[11] Pan X, Li Z, Huang X (2019). Molecular basis for pore blockade of human Na+ channel Na(v)1.2 by the mu-conotoxin KIIIA. Science.

[12] Li X, Xu F, Xu H (2022). Structural basis for modulation of human NaV13 by clinical drug and selective antagonist. Nat Commun.

[13] Pan X, Li Z, Zhou Q (2018). Structure of the human voltage-gated sodium channel Nav1.4 in complex with beta1. Science.

[14] Li Z, Jin X, Wu T (2021). Structural basis for pore blockade of the human Cardiac Sodium Channel Nav 1.5 by the Antiarrhythmic Drug Quinidine*. Angew Chem Int Ed Engl.

[15] Li Z, Jin X, Wu T (2021). Structure of human Nav1.5 reveals the fast inactivation-related segments as a mutational hotspot for the long QT syndrome. Proc Natl Acad Sci USA.

[16] Shen H, Liu D, Wu K, Lei J, Yan N (2019). Structures of human Nav1.7 channel in complex with auxiliary subunits and animal toxins. Science.

[17] Mantegazza M, Curia G, Biagini G, Ragsdale DS, Avoli M (2010). Voltage-gated sodium channels as therapeutic targets in epilepsy and other neurological disorders. Lancet Neurol.

[18] Herranz JL, Armijo JA, Arteaga R (1988). Clinical side effects of phenobarbital, primidone, phenytoin, carbamazepine, and valproate during monotherapy in children. Epilepsia.

[19] Aldenkamp AP, Vermeulen J (1995). Phenytoin and carbamazepine: differential effects on cognitive function. Seizure.

[20] Swain NA, Batchelor D, Beaudoin S (2017). Discovery of Clinical Candidate 4-[2-(5-Amino-1H-pyrazol-4-yl)-4-chlorophenoxy]-5-chloro-2-fluoro-N-1,3-thiazol-4 -ylbenzenesulfonamide (PF-05089771): design and optimization of diaryl ether aryl sulfonamides as selective inhibitors of NaV1.7. J Med Chem.

[21] McCormack K, Santos S, Chapman ML (2013). Voltage sensor interaction site for selective small molecule inhibitors of voltage-gated sodium channels. Proc Natl Acad Sci USA.

[22] Johnson JP, Focken T, Khakh K (2022). NBI-921352, a first-in-class, NaV1.6 selective, sodium channel inhibitor that prevents seizures in Scn8a gain-of-function mice, and wild-type mice and rats. Elife.

[23] Ahuja S, Mukund S, Deng L (2015). Structural basis of Nav1,7 inhibition by an isoform-selective small-molecule antagonist. Science.

[24] Kim S, Chen J, Cheng T (2021). PubChem in 2021: new data content and improved web interfaces. Nucleic Acids Res.

[25] Mendez D, Gaulton A, Bento AP (2019). ChEMBL: towards direct deposition of bioassay data. Nucleic Acids Res.

[26] Gilson MK, Liu T, Baitaluk M, Nicola G, Hwang L, Chong J (2016). BindingDB in 2015: a public database for medicinal chemistry, computational chemistry and systems pharmacology. Nucleic Acids Res.

[27] UniProt C (2021). UniProt: the universal protein knowledgebase in 2021. Nucleic Acids Res.

[28] Burley SK, Bhikadiya C, Bi C (2021). RCSB Protein Data Bank: powerful new tools for exploring 3D structures of biological macromolecules for basic and applied research and education in fundamental biology, biomedicine, biotechnology, bioengineering and energy sciences. Nucleic Acids Res.

[29] Varadi M, Anyango S, Deshpande M (2022). AlphaFold Protein Structure Database: massively expanding the structural coverage of protein-sequence space with high-accuracy models. Nucleic Acids Res.

[30] Pavlov D, Rybalkin M, Karulin B, Kozhevnikov M, Savelyev A, Churinov A (2011). Indigo: universal cheminformatics API. J Cheminform.

[31] Smith JJ, Alphy S, Seibert AL, Blumenthal KM (2005). Differential phospholipid binding by site 3 and site 4 toxins. Implications for structural variability between voltage-sensitive sodium channel domains. J Biol Chem.

[32] Field LM, Emyr Davies TG, O'Reilly AO, Williamson MS, Wallace BA (2017). Voltage-gated sodium channels as targets for pyrethroid insecticides. Eur Biophys J.

[33] Xiong G, Wu Z, Yi J (2021). ADMETlab 2.0: an integrated online platform for accurate and comprehensive predictions of ADMET properties. Nucleic Acids Res.

[34] Todsen WL (2014). ChemDoodle 6.0. J Chem Inf Model.

[35] Rego N, Koes D (2015). 3Dmol.js: molecular visualization with WebGL. Bioinformatics.

[36] Sadybekov AA, Sadybekov AV, Liu Y (2022). Synthon-based ligand discovery in virtual libraries of over 11 billion compounds. Nature.

[37] Wang P, Wadsworth PA, Dvorak NM (2020). Design, synthesis, and pharmacological evaluation of analogues derived from the PLEV Tetrapeptide as protein-protein interaction modulators of voltage-gated sodium channel 1.6. J Med Chem.

